# Can current preclinical strategies for radiopharmaceutical development meet the needs of targeted alpha therapy?

**DOI:** 10.1007/s00259-024-06719-5

**Published:** 2024-04-27

**Authors:** Janke Kleynhans, Thomas Ebenhan, Frederik Cleeren, Mike Machaba Sathekge

**Affiliations:** 1 Laboratory for Radiopharmaceutical Research, Department of Pharmaceutical and Pharmacological Sciences, KU Leuven, Leuven, 3000 Belgium; 2 Department of Nuclear Medicine, University of Pretoria, and Steve Biko Academic Hospital, Pretoria, 0001 South Africa; 3 Department of Nuclear Medicine, Steve Biko Academic Hospital, Pretoria, 0001 South Africa; 4 Preclinical Imaging Facility, Nuclear Medicine Research Infrastructure, Pretoria, 0001 South Africa

**Keywords:** Targeted alpha therapy, Preclinical evaluation, Actinium-225, Theranostics, Lead-212, Astatine-211

## Abstract

Preclinical studies are essential for effectively evaluating TAT radiopharmaceuticals. Given the current suboptimal supply chain of these radionuclides, animal studies must be refined to produce the most translatable TAT agents with the greatest clinical potential. Vector design is pivotal, emphasizing harmonious physical and biological characteristics among the vector, target, and radionuclide. The scarcity of alpha-emitting radionuclides remains a significant consideration. Actinium-225 and lead-212 appear as the most readily available radionuclides at this stage. Available animal models for researchers encompass xenografts, allografts, and PDX (patient-derived xenograft) models. Emerging strategies for imaging alpha-emitters are also briefly explored. Ultimately, preclinical research must address two critical aspects: (1) offering valuable insights into balancing safety and efficacy, and (2) providing guidance on the optimal dosing of the TAT agent.

**Supplementary Information:**

The online version contains supplementary material available at 10.1007/s00259-024-06719-5.

## Introduction

Radiotheranostics, a worldwide expanding clinical procedure in oncology, combines medical nuclear imaging with targeted radionuclide therapy. This strategy involves mostly the systemic intravenous administration of a radiopharmaceutical. The radiopharmaceutical consists out of a vector with high affinity and selectivity for the target tissue and either diagnostic or therapeutic radionuclide [1, 2]. Single photon emission computed tomography (SPECT) and positron emission tomography (PET) are the two molecular imaging techniques that are available in nuclear medicine.

Within the field of radiotheranostics, targeted alpha therapy (TAT) aims to harness the high cytotoxic payload of alpha-emitting radionuclides to treat cancer based on unique tumor cell targets. Alpha particles have the desired short penetration range within the tissue which allows focused endogenous radiation of a restricted region of interest, with minimal damage to surrounding tissue [3, 4]. The high linear energy transfer (LET), leading to a high number of ionizations per unit path-length, allows for the increased localized cytotoxicity of TAT which has been proven beneficial in (pre)clinical studies. In contrast, beta particle emitters such as lutetium-177, travel more distance in tissue before depositing their energy demonstrating a longer path length and lower LET.

**Fig. 1:**
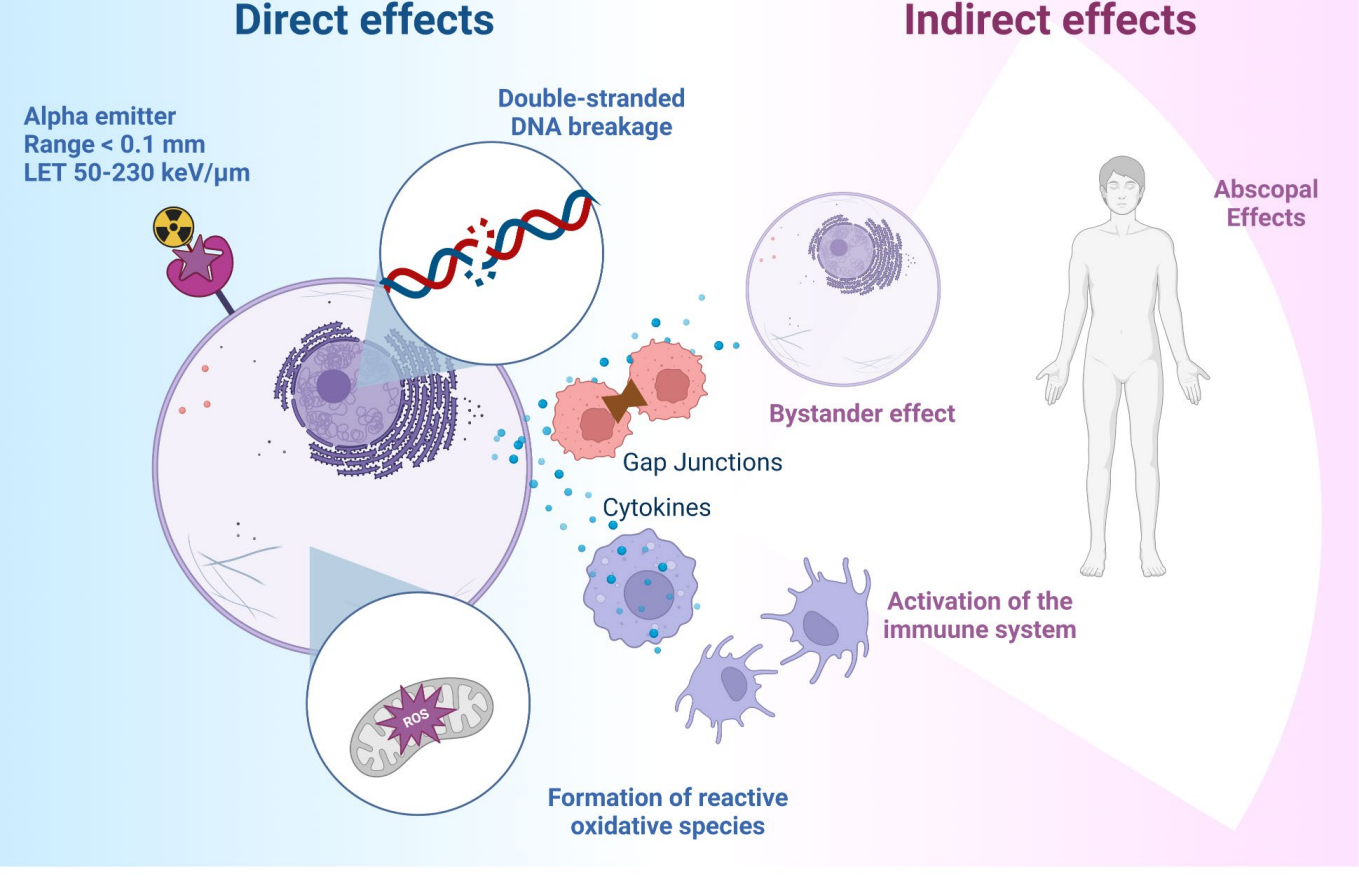
Direct and indirect effects associated with targeted alpha radiation (Created with Biorender.com)

The high LET of alpha particles causes direct damage to DNA by double-stranded DNA breaks which are more difficult to repair than single-stranded DNA breaks or causes indirect damage through free radicals generated by water radiolysis (Fig. 1). Direct or indirect DNA damage is the main mechanism of cytotoxicity, but other effects also contribute to the overall cell-killing efficiency of alpha-particles. This includes the radiation-induced bystander effect as well as the abscopal effect from the activation of a radiation-induced immune response [5]. It is also important to note that, in contrast to beta-particle therapy, the toxicity of alpha-particles is independent of cellular oxygenation due to a lower reliance on the formation of reactive oxygen species as mechanism of toxicity. The presence of hypoxia in tumor tissue is therefore less hindering for therapy with TAT agents. Alpha emitters therefore have higher biological effectiveness compared to beta particle emitting radionuclides [6, 7].

Currently, Xofigo^®^ ([^223^Ra]RaCl_2_) is the sole radiopharmaceutical containing an alpha emitting radionuclide, approved by the FDA (United States Food and Drug Association) [8]. However, a significant number of clinical trials are being conducted to prove the efficacy and value of TAT procedures in oncology. The complete list of relevant clinical trial investigations is provided in Table S1. Excluding trials involving [^223^Ra]RaCl_2_ (a total of 122 studies since 2005 till current), a combined 39 alpha-emitter-based clinical trials have been registered since 2005 on the ClinicalTrial.gov website. Actinium-225 leads the clinical trial spectrum with a total of 23 new trials registered until 2023, with PSMA (prostate-specific membrane antigen) and SSTR2 (somatostatin receptor 2) the most notable therapeutic targets. Most registered trials are either covering Phase I or the combination of Phase I/II trials clinical investigations. The exception is [^225^Ac]Ac-DOTA-TATE which is currently in the recruitment stage of a Phase III clinical trial [9]. Other clinical investigations using lead-212 and astatine-211 are also gaining momentum [10–16]. Alpha emitters are more resistant to adaptive mechanisms that might render other therapeutic radiopharmaceuticals less efficient. To date, no cellular mechanisms of resistance have been described for TAT [13–16].

To allow TAT procedures to possibly enter mainstream application in oncology, preclinical investigations must be appropriately balanced to rigidly assess the most viable TAT candidate radiopharmaceuticals in a resource-effective way. It is important at this stage to make a clear distinction between the approach to be followed in evaluating conventional chemotherapeutic cancer therapies, i.e., research concerning cytotoxic chemical or biological agents, and those pertinent to therapies harnessing the potency of radiation to induce cytotoxic effects. Sgouros and co-workers [17] published a thought-provoking commentary on the role of preclinical models in facilitating the clinical implementation of radioligand-based therapies. It is often proposed that changes in tumor growth in a preclinical model are one of the success criteria for efficacy. It is important to consider the limitations of xenografts as a preclinical model, especially subcutaneous models. As such, more advanced models should be considered such as orthotopic models and patient derived xenografts.

Radiotheranostics has sparked increasing interest and gained importance in parallel to the growth in molecular imaging and personalized medicine. The diagnostic partner radiopharmaceutical can assist with patient selection, prediction of response and toxicity, and determination of prognosis. Direct imaging strategies using alpha particle-emitting radionuclides is also being investigated.

Due to its rapid development and research output provoked by new, clinically relevant targets and interest from pharmaceutical industry, it is currently challenging to identify common rules that could enhance the success of targeting strategies of TAT. As radiotheranostic approaches are ever-expanding, the importance of the preclinical setting deserves more attention regarding the accurate evaluation of emerging TAT agents.

TAT research focuses on multiple aspects of advancement including target discovery, radionuclide production, radiochemistry, infrastructure platforms, quality control methods, dosimetry, monotherapy and the use of combination therapies [18].

This article will focus on reviewing the capabilities of the preclinical setting towards the tailored requirements for TAT to better support prospective clinical trials for new radiotheranostic pairs. The value and status of the available tools to realize preclinical evaluation of TAT are presented followed by promising preclinical examples and the overview of the regulatory framework for clinical translation. The relevance of preclinical research addressing efficacy through alpha-particle radiation dose delivery and safety for systemic administration are discussed.

## The tools for the success of TAT design

To ensure that the developed TAT agent has the most optimal chances for clinical translation, the unique interplay between the molecular target, targeting vector and the characteristic of the incorporated radionuclide must be considered. There are current fashionable targets dictated by clinical need, but the chosen vectors must address the need in the most effective way possible. Tailoring the ideal match between the targeting vector and the radionuclide to deliver the alpha therapy payload is an important criterion to consider during TAT design.

### Current tumor cell targets for TAT

The targeting of prostate-specific membrane antigen (PSMA) for imaging and therapy in prostate cancer has showed success in the nuclear medicine clinic. Not only for the management and staging of patients, but also for therapy. This radiopharmaceutical has very attractive qualities for TAT, being overexpressed in prostate cancer leading to high specificity. As mentioned previously, many actinium-225 based PSMA agents are currently in various clinical trial phases. Despite the success of ***PSMA*** targeting radiopharmaceuticals, there is still room for optimization and a lot of preclinical research is focusing on bettering the technology. A main research priority is the reduction of off-target irradiation in the salivary glands. The development of xerostomia is a dose-limiting factor in patients and influences quality of life. Xerostomia leads to poor nutrition, gingivitis and symptoms of anxiety and depression. Multiple efforts are underway to develop novel PSMA targeting constructs with different chemical structures that could lead to lower or no accumulation in salivary glands [19]. The incorporation of radionuclides other than actinium-225, such as astatine-211 and lead-212, is also being investigated [20, 21]. Increasing circulation time to optimize tumor accumulation is also being investigated but potential increased toxicity needs to be considered [22, 23].

***SSTR2*** is targeted in the treatment of neuro-endocrine tumors. Indeed, this application of TAT has progressed the furthest in the clinic with a phase III clinical trial registered for [^225^Ac]Ac-DOTA-TATE [24]. Neuroendocrine tumors (NETs) are a group of rare tumors that arise from cells of the neuroendocrine system. These tumors can develop in various organs throughout the body, with the most common sites being the gastrointestinal tract, pancreas, and lungs [25]. Currently, a large amount of research effort is geared towards the development of SSTR2 receptor antagonists since there is some evidence that this might lead to more optimal therapeutic efficacy. This is to the fact that SSTR2 antagonists seems to recognize more binding sites on receptors resulting in higher accumulation at the target site, despite poor internalization. It is however still to be determined if this could lead to a more favorable therapeutic index or if this will lead to potentially higher toxicity in low SSTR2 expressing healthy tissues [26]. The higher accumulation of these TAT agents in SSTR2 expressing lesions might lead hopefully to therapy that will eradicate the tumors rather than just partial responses [27]. One should also consider the potential toxic effects of recoiling daughters in the case of ^225^Ac-labeled SSTR2 antagonists as the radiopharmaceutical will localize on the cell membrane in contrast to SSTR2 agonists that are being internalized, more research is highly needed in this field to make the correct conclusions.

The **tumor microenvironment** is viewed as an extremely attractive target for TAT as this could provide a pan-tumor agent that can be used to treat many cancers. New radiotheranostic approaches are investigated that look at targets within the tumor microenvironment such as blood vessels, cancer-associated fibroblasts, the stromal matrix and immune cells. However, since the targeting of the TAT agents is done at the tumor microenvironment in this case, and not the cancer cells themselves, it is critical that efficacy should be proven and that the path length of alpha emitters is adequate to irradiate the whole of the target area. Some prominent targets investigated in this group include angiogenesis (mostly RGD-based) and FAP (fibroblast activating protein) targeting molecules. Promising preclinical examples are astatine-211-based FAPI ligands and angiogenesis targeting astatine-211 RGD constructs [28–30].

**HER 2** is most notably overexpressed in certain types of aggressive breast cancer, but also in some other malignancies. Treatment for this aggressive malignancy is still not adequate and the availability of a TAT agent is urgently needed. Actinium-225-DOTA antibody conjugates [31] are actively investigated for efficacy.

A revisiting of **bone-seeking** agents is also getting attention with radium-223 as the main agent being investigated clinically. There is still much uncertainty regarding the ideal time during disease progression, combinations with other therapies and dosing schedules for the administration of radium-223. Alternative alpha emitters containing bone agents such as [^225^Ac]Ac-DOTA-zolendronate have also been investigated [32].

**Blood cancers** are an important area of investigation for TAT agents, mostly involving alpha radioimmunotherapy agents [33, 34]. In this group, the investigation of CXCR4 targeting (with as example Pentixather) is also an active area of research. Interestingly, a phase 1 trial with ^212^Pb-pentixather in patients with atypical lung carcinoids and neuroendocrine carcinomas is being registered, and ^203^Pb-labeled Pentixather will be used to assess CXCR4 expression levels and for dosimetry purposes [35].

**PARP inhibitors** targeting the poly (ADP-ribose) polymerase (PARP) enzyme are also investigated as targeting vectors for TAT agents. This enzyme is involved in DNA repair pathways and is upregulated in many cancer types. Not only would a diagnostic agent quantifying this disease mechanism be beneficial, but it also provides a valid target for theranostic TAT applications [36]. In fact, many more targets are investigated for TAT and many excellent, recent, in-depth reviews have been published on this topic [5, 9–11, 16].

### Ideal vectors for the safe delivery of TAT

**Vector Design** is critical for the optimal delivery of TAT. The biochemical vector is responsible for the selective interaction with the target tissue, by vector design leading to a higher concentration of the radionuclide in the target tissue compared to non-target tissue. This makes selective irradiation of the target cells possible [37]. As most vectors have a saturable pharmacological, immunological, or metabolic interaction with their target, only tracer amounts of the radiopharmaceutical must be administered to avoid saturation of the target [38]. The retention in the target tissue can be due to a reversible interaction such as affinity-based receptor binding, governed by equilibrium association and dissociation. After binding to the target, the tracer may however be internalized and retained in the cell, leading to pseudo-irreversible kinetics. Internalization in the cell can also be an advantage when in vivo generators are used such as ^225^Ac-labeled radiopharmaceuticals. It is assumed that recoiling daughters may be trapped in the target cells, which would not be the case if the vector remains on the cell membrane allowing recoiling daughters to escape the target tissue, leading to potential toxicity of healthy tissue [38, 39]. For TAT, the most used vector molecules can be categorized into small molecules, peptides, and proteins such as antibodies and antibody fragments [40, 41].

***Small molecules***, occurring in a large variety, can be used as vector molecules, for example using biochemicals such as amino acids, fatty acids, and nucleosides. In contrast to larger molecules, these small molecules have excellent tumor penetration capabilities and mostly a fast excretion profile [42]. A challenge with small molecules is the fact that after conjugation with a bifunctional chelator and radiolabeling, they still need to demonstrate an affinity for the cellular target to ensure a sustained tumor accumulation [43].

***Peptides*** with affinity to cancer cell-specific receptors are often suggested as vector molecules for theranostic radiopharmaceutical applications. Indeed, the overexpression of many peptide receptors on human tumor cells compared to normal tissues has made certain receptors already attractive targets for peptide-receptor radionuclide therapy (PRRT) [44]. Peptides also show rapid diffusion into target tissue due to their low molecular weight. Moreover, upon binding of the radiolabeled peptide analogue (RPA), the receptor-RPA complex is often internalized, allowing long retention of radioactivity in tumor cells which is a fitting requirement for TAT to be successful, especially in combination with longer lived alpha-emitting radionuclides such as actinium-225 [45]. Further, peptides are easily synthesized using an automated peptide synthesizer and most peptides can tolerate harsh radiolabeling conditions such as high temperature and low pH [46, 47]. Finally, known peptide sequences of amino acids can be modified to decrease their catabolic rate once exposed to the in vivo setting (modifications can include incorporation of D-amino acids, the use of pseudo-peptidyl bonds and cyclic peptide formations) [48]. Other advantages of using peptides as vector molecules are their favorable pharmacokinetics characterized by rapid clearance from the blood pool and non-target tissue, and often the absence of liver clearance followed by hepatobiliary excretion. High concentration in the target tissue is often observed, however, this depends on the target expression level, the basal expression of the target in healthy tissues, and the pharmacokinetics and affinity of the peptide [49, 50]. An unfitting issue often associated with the use of radiolabeled peptides is their high renal uptake and kidney retention, particularly for TAT the risk may occur for potential radio-nephrotoxicity [51, 52].

***Proteins*** can also be used as vector molecules for radiopharmaceuticals if radiosynthetic procedures can abstain from high temperatures and organic solvents, i.e., mild radiolabeling conditions in aqueous (often pH neutral) medium needs to be applied using optimized chelators for each specific radionuclide [53]. Intact monoclonal antibodies (mAb) are considered good candidates for TAT because they provide a versatile platform of probes with outstanding affinity and specificity towards a variety of tumor-unique antigens. Their large size (150 kDa), which excludes glomerular filtration, combined with Fc-mediated catabolism escape, results in a circulation of several days to weeks in blood. In addition, as the list of approved mAbs and antibody drug conjugates (ADCs) by FDA and EMA is constantly increasing, this creates opportunity to use the same mAbs in an efficient way also for TAT applications. In general, a high target-to-background ratio can only be obtained several days after intravenous injection of the radiolabeled mAbs [54, 55]. Therefore, the use of short-lived alpha particle-emitting radionuclides is not an option for systemic administration as mainly the bone marrow and healthy organs would receive the highest dose [56]. Only long-lived radionuclides will result in sufficient high tumor-to-background dose [57]. Further, the use of actinium-225 radionuclides in combination with long-circulating vector molecules might cause concerns, as in theory, the recoiling daughters can escape the vector molecule during circulation time, resulting in irradiation of healthy tissue due to the unwanted release of daughter radionuclides. Efforts are made to address this phenomenon with more in-depth dosimetry studies to quantify this potential problem [58, 59].

**Fig. 2:**
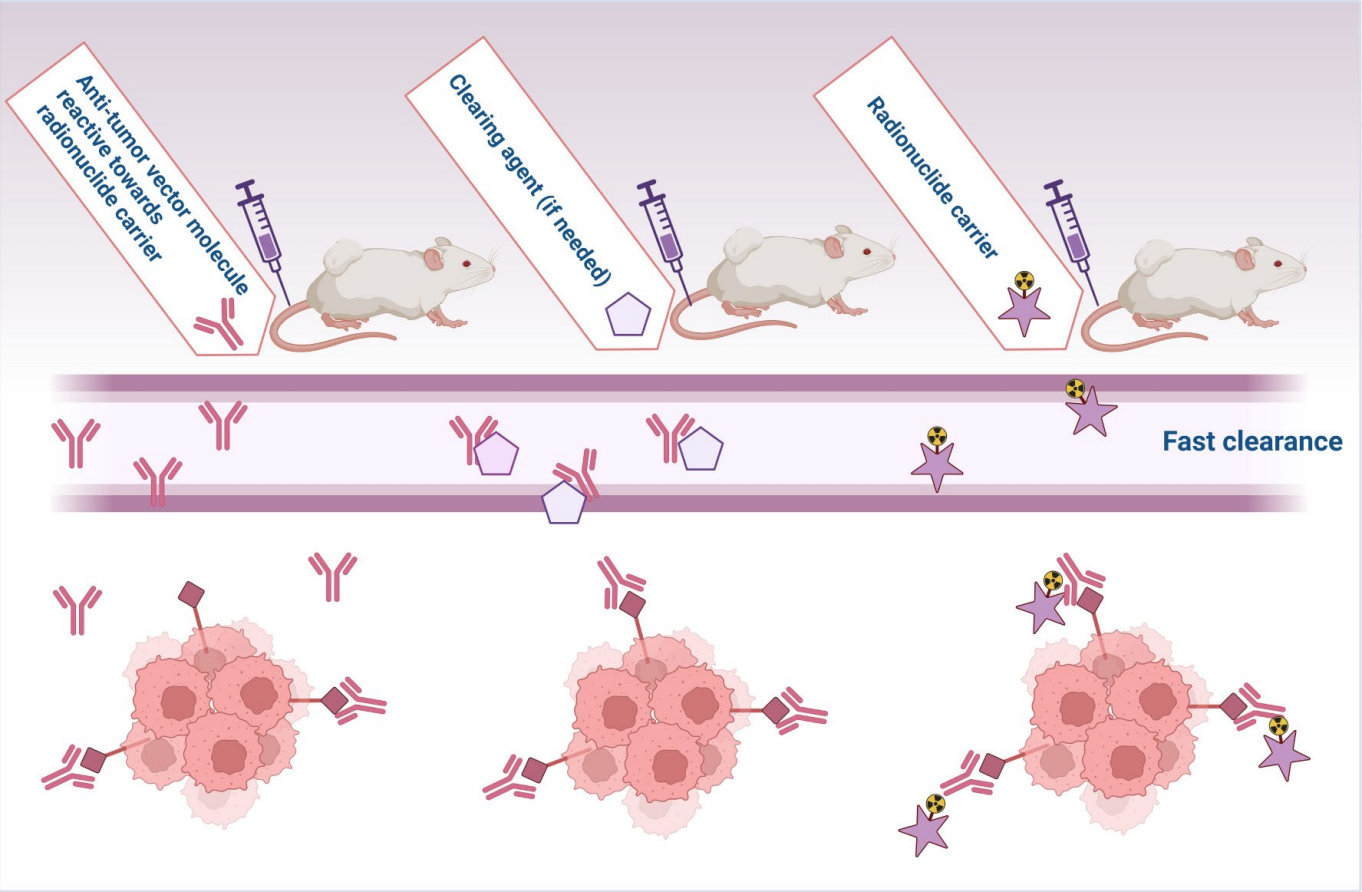
Visual presentation of the pre-targeting principle with referencing Cheal et al. [60] and Poty et al. [61] (Created with Biorender.com)

Also of promise is the use of pre-targeting strategies where the desirable target-to-background ratio of antibodies are combined with the ideal pharmacokinetics of small molecules. Firstly, a vector molecule that has affinity for the target and can react in vivo with the radioactive carrier is injected and accumulates in the target tissue. Unbound circulating vector molecules can be removed by a clearing agent if needed, whereafter a fast-clearing radionuclide carrier is injected to react in vivo with the vector molecule and thus accumulates at the tumor tissue (Fig. 2) [60, 61]. This approach is from a logistic point of view more challenging due to the 2- or 3 step approach but can result in an increased therapeutic index of TAT.

***Advancements in biotechnology*** have led to the bioengineering of many vector molecules with shorter biological half-lives. Often these bioengineered molecules can be labeled using site-specific radiolabeling methods. Because of the smaller size, these engineered proteins are smaller than the cut-off rate for glomerular filtration resulting in much faster clearance from non-target tissue. Whilst this technology can be applied to all TAT strategies, it can be particularly useful to allow radionuclides with shorter half-lives (such as bismuth-213: 45.6 min) to reach the target in time for the release of the radiation payload. As with peptides, an issue is their high uptake and retention by the kidneys causing potential radio-nephrotoxicity [62–65].

### Alpha particle-emitting radionuclides – value and availabilities

As reported exhaustively, the obvious bottleneck for the efficient development of TAT procedures is the current availability of alpha particle-emitting radionuclides accounting for future feasibility [66, 67]. As such, the availability of an alpha particle-emitting radionuclide (influenced by infrastructure & raw material) and the economic viability thereof becomes of major importance when selecting what alpha particle-emitting radionuclides to pursue for current research. If clinical translation is the desired outcome, it is illogical to pursue TAT developments that, however elegant they may be, would be too expensive and scarce to become globally available.

Currently pursued alpha particle-emitting radionuclides in TAT are provided in Table 1. In 2021 Eychenne and co-workers identified the so-called “hopeful eight” alpha particle-emitting radionuclides, namely: actinium-225, astatine-211, bismuth-212 and 213, lead-212, radium-223, terbium-149 and thorium-227 [68]. An in-depth discussion on the production and supply of alpha particle-emitting radionuclides for TAT was recently reported [69]; however, the value and current constraints for each alpha particle-emitting radionuclide will hereby be given below.

**Table 1:** General characteristics of alpha emitters investigated for TAT, ranked from longest physical half-life to shortest [6, 15, 16]

Radionuclide	Physical half-life	Production	E_α max_ (Mev)	R_av_
Thorium-227	18.7 days	Produced by the decay of actinium-227	5.87/26.70	50–70 μm
Radium-223	11.4 days	Produced by the decay of actinium-227, thorium-227 and radium-226	5.87/26.70	45 μm
Actinium-225	9.9 days	Produced from a thorium-229 generator, irradiation of thorium-232 with high energy photons, irradiation of radium-226 with medium energy protons or photons	5.83/27.62	61 μm
Lead-212	10.64 h	Produced by thorium-228 and radium-224 decay in generator format.	5.69/27.54	40–100 μm
Astatine-211	7.21 h	Produced by the cyclotron irradiation of bismuth-209 with alpha particles.	5.87	60 μm
Terbium-149	4.1 h	Most promising method is through the proton induced direct nuclear reaction with gadolinium-152.	3.97	25 μm
Bismuth-212	60.6 min	Produced by radium-224 generator.	8.5	40–100 μm
Bismuth-213	45.6 min	Produced by actinium-225 generator.	8.4	84 μm

**Actinium-225** as recently highlighted [70], will become more widely available than even beta-minus emitters lutetium-177 and terbium-161, utilizing new accelerator production strategies. This results in actinium-225 being an extremely attractive alpha-emitting radionuclide to pursue for research and development, despite a less optimal decay scheme resulting in recoiling daughter nuclides. A major advantage of ^225^Ac-radiopharmaceuticals from an industrial point of view is the relative long half-life of ^225^Ac, allowing centralized production and distribution. The logistics and infrastructures are in place from the current pipeline of ^177^Lu-labeled radiopharmaceuticals which have paved the way for TAT.

The stability of actinium-225-based radiopharmaceuticals is of major importance and the decay scheme (4 alpha decays) influences the design and quality control of these radiopharmaceuticals [71]. Validated quality control methods and acceptance criteria needs to be in place, also for stability evaluation of the final product. One should not only assess if the radionuclide is still attached to the vector molecule but also assess the intactness of the vector molecule itself and its functionality. Optimized formulation buffers need to be available to guarantee sufficient shelf-life if centralized production and distribution is considered. The detection and monitoring of long-lived radionuclides that might be present from the production processes is also an important topic that needs to be handled with care. Such an example is actinium-227 produced in an amount of less than 0.3% during the accelerator production of actinium-225 via thorium-232 irradiation. In such a case care needs to be taken to address the additional burden on waste management of long-lived radionuclides. Other alleviating processes include the optimizing production methods to reduce the formation of these long-lived radionuclides, removal from these radionuclides during purification processes (e.g. only using radium radioisotopes from the throrium-227 production) and ensuring through dosimetry calculations that the radiation from small percentage long-lived radioisotopes would not influence the patient negatively [70, 72].

With the advent of increased actinium-225 production and availability, this will naturally lead to the increased availability of bismuth-213. **Bismuth-213** is obtained from a generator loaded with actinium-225 as the parent radionuclide. Currently, it is the least featured alpha particle-emitting radionuclide in registered clinical trials; shortages in production and its short physical half-life, necessitating on-site production of the ^213^Bi-radiopharmaceuticals; are currently limiting the application [72, 73]. Bismuth-213 radiopharmaceuticals are administered in high activities (e.g. 2.1 GBq per cycle for [^213^Bi]Bi-Substance P) and therefore also need a high amount of actinium-225 to be loaded on the generator which is currently a restraint for clinical productions. For bismuth-213 TAT to be effective, novel radiopharmaceuticals must be designed that demonstrate high tumor load due to rapid kinetics to warrant an optimal delivery of cytotoxic radiation [74]. The ideal bismuth-213 radiopharmaceutical will therefore demonstrate very fast clearance, no kidney retention and high tumor accumulation. Alternatively intra-tumor delivery of radiopharmaceuticals rather than systemic delivery could be investigated.

**Astatine-211** has limited availability and the radiochemistry is challenging, which has hampered its clinical translation for TAT. Whilst the production and purification processes are inexpensive, globally there are a limited number of accelerators capable of producing the required 28 MeV alpha particle beam with adequate intensity levels to master a scaled production [75]. The relatively short physical half-life makes the logistics challenging for centralized production and distribution, but as high quantities of ^211^At-radiopharmaceuticals can be produced, distribution should be feasible as it is now also possible for ^18^F-labeled diagnostic tracers. This radionuclide is very attractive in a clinical point of view due to its beneficial half-life and single alpha emitter decay process, and it is hoped that problems with availability and complex radiochemistry will be solved soon.

**Terbium-149** is viewed as an attractive alpha-particle emitter due to its near-ideal decay properties. It features a moderate physical half-life, with the absence of problematic decay daughters, and a low alpha-energy (3.97 MeV). However, to date, production of this radionuclide could only yield doses sufficient for preclinical evaluation. Further, the short physical half-life makes central production and distribution challenging. Worldwide efforts are geared toward the production of terbium-149 in higher quantities to support exploratory clinical trials in humans, but progress is slow at this moment [69, 76].

**Lead-212** is considered “hopeful” because it is a beta-emitter that provides decay-based secondary alpha radiation inside the body (i.e., often explained as an in vivo generator). Using lead-212 TAT could indeed be an elegant approach as it combines beta-minus radiation creating two short-lived alpha particle emissions. It may become more widely available due to an extremely affordable radionuclide production with a proposed near-infinite capacity. It is important to note that unlike longer-lived radionuclides, lead-212 would be made available by a generator system necessitating on-site GMP production and quality control of lead-212 containing radiopharmaceuticals. Many efforts are geared towards the production of GMP compatible generators, also considering radiation exposure of operators. Caution is warranted due to the less ideal radiation dosimetry due to the high-energy gamma-emission of its radionuclide daughter thallium-201 as well as the partial release of bismuth-213 from the currently used chelator systems [77]. Given these limitations, efforts are being made to alleviate these weaknesses [77].

**Thorium-227**, the progenitor nuclide of radium-223, offers promise as a wider-ranging alternative due to the availability of efficient chelators [78]. For the past decade, this alpha particle-emitting radionuclide has attracted attention for several forms of systemic radionuclide therapy. The longer physical half-life of thorium-227 makes it suitable for the treatment of hematologic malignancies and targeted radioimmunotherapy. Again, the problematic decay chain (5 alpha decays) might hamper straightforward clinical translation [79, 80].

**Radium-223**, although clinically useful for alpha-therapy, is not included in this review due to the lack of good bifunctional chelators for radium, making TAT with radium-223 not possible at this moment [81, 82]. Some attempts have been made to incorporate radium-223 in TAT nanoparticle systems [83, 84].

It seems that TAT research the coming years will be focused on the two radionuclides with the most abundant availability, being actinium-225 and lead-212. However, astatine-211 research and production are also being ramped-up and might also play an increasing important role. It is also important to note that long-lived radionuclides allowing centralized production is ear-marked for additional infrastructure investment, since this will offer comparable logistic supply chain management with successful technologies such as lutetium-177. It is expected that automatization of radiochemistry processes and radiopharmaceutical production will also become more and more introduced. A vast body of research addressing the upscaling of production concerning alpha particle-emitting radionuclides is currently being realized. The latter aspects will allow the effective translation and utilization of alpha radionuclides which is still very much a limited resource at this moment [69].

### Requirements for animal models to support TAT of cancers

Animal drug testing before human exposure can be considered a critical development step to ensure safety and efficacy. However, preclinical testing falls short of expectations, with only a third of preclinically approved drugs entering clinical trials. Drugs that passed preclinical testing has a failure rate of 85% (all phases included). Even therapeutic agents that make it past phase III only demonstrate a 50% success rate [85, 86]. One factor could be the unfitting use of cell line models and animal models in the preclinical research settings which may lack in reflecting the physiological situation of humans. Differences in size and physiology as well as variations in the homology of targets between mice and men are inevitable contributors to translational limitations. This aspect cannot be ignored, for the development of new radiotheranostic pairs for TAT, in particular. For example, the authors recently reviewed the development of radiotheranostics for human glioblastoma *multiforme* (GBM) and have illustrated that the available rodent tumor models are suboptimal simulations of the features of the original GBM tumors [87]. Another review, focusing on the mouse model design, concludes similar weaknesses and shortcomings, and highlights the need to improve the predictive power of preclinical cancer models [88].

A possible solution may present itself from the tissue bioengineering sector which provides exponential improvements regarding the way cell cultures are grown, resulting in either a 3D-spheric shape or complete cancer organoids with or without co-cultured immune cells. These models might have a larger relevance for the preclinical setting to address the needs of TAT compared to standard in vitro cell experiments [89]. For all in vitro efficacy experiments it is important to include proper controls to exclude medium effects, being unbound radiopharmaceuticals in the medium that irradiate cells.

Patient-derived xenograft (PDX) models have gained popularity in cancer research due to their enhanced representation of the tumor heterogeneity, the more accurate composition of the tumor microenvironment, retention of the cellular complexity, cytogenetics, and stromal architecture. In these models, tumor tissue samples from patients are taken and implanted into the immune-compromised mice. A sequence of samplings and implantations follows to allow for the stabilization of the PDX model. PDX models now have been identified as a powerful tool for determining cancer characteristics, developing new treatments, and predicting drug efficacy. Alignment with noninvasive imaging may create a powerful alliance for future research investigations. A well-established PDX model maintains all the characteristics of the patient tumor but is enhanced by real tumor-immune interactions provided by the host. It is important to note that PDX models can be quite complex to implement and methods of tumor acquisition, treatments received by the patient, sampling size, and the origin of the tumor (primary or metastatic) can all influence the success rate of the PDX models [55, 90, 91].

The use of PDX models in TAT investigations has been more limited than other models [92–94]. PDX models have been applied to evaluate these agents in an environment mimicking the cancer heterogeneity prevalent in clinical trials. The variability in efficacy is almost exclusively correlated with the variability of target expression across different patient-derived tumors [95]. In a few publications, tissue allografts (syngeneic or orthotopic transplants) from mice have been used to explore the efficacy and survival benefit of TAT [96–99]. However, no rationale has been provided in these publications for this choice. It seems that allografts do have a particular application in the evaluation of immune checkpoint inhibitors and other combination therapies that might need a more natural disease profile to the host [98]. For combination therapy it is therefore important to have a mouse model with an intact mouse immune system. It is also important to note that immunodeficient mice have differences in radiosensitivity and this can influence the survival of the animals during efficacy studies. For instance, SCID mice are known to be hypersensitive towards radiation which makes them less suitable for efficacy studies [100].

In general, for all in vivo efficacy studies, study design is important, control groups need to be included and reporting should follow the ARRIVE guidelines. To evaluate the therapeutic efficacy, the therapeutic radiopharmaceutical can be injected in a single dose, or in a repeated dose interval scheme. Tumor growth can be evaluated before and during treatment using the diagnostic sister radiopharmaceutical or with bioluminescence imaging if luciferase expressing cells are used or physically using a Vernier caliper in the case of subcutaneous models. Further, [^18^F]FDG PET scans can be performed to quantify functional tumor tissue. Finally, weight, survival (Kaplan-Meier curves), and toxicity to the liver and kidneys should be evaluated and blood values should be monitored.

Just like in animals, a cornerstone of theranostics is to study and assess the presence of the tumor target in a patient through the imaging procedure by way of involving the diagnostic counterpart. The use of a diagnostic counterpart therefore already alleviates the issue concerning variation in patient disease profiles and is an excellent example of personalized medicine.

## Preclinical research examples for TAT-tailored imaging strategies

**Fig. 3:**
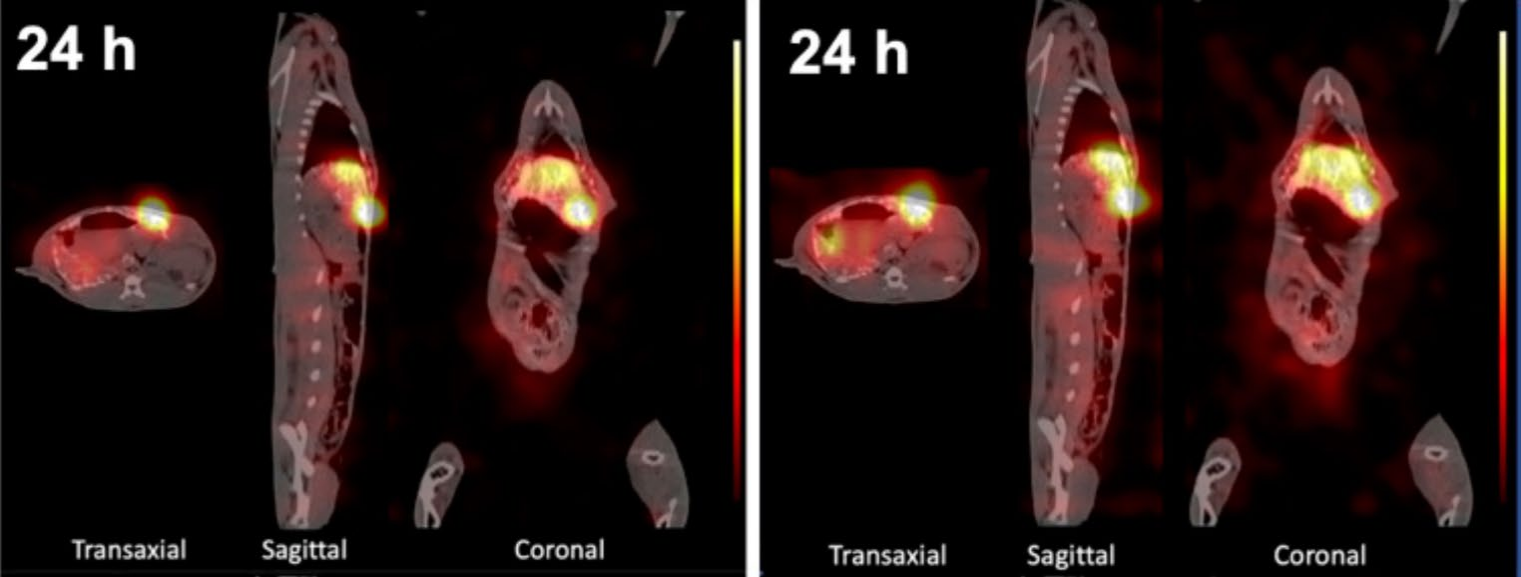
Representative nuclear imaging of a rabbit bearing VX2 hepatic tumor xenograft. The micro-SPECT/CT images were acquired and reconstructed using the francium-221 energy window (left) and bismuth-213 energy window (right), respectively. Reprinted with permission [101]

Performing preclinical imaging using alpha particle-emitting radionuclides is often intricate, cumbersome and costly or simply not yet adequately described in literature. The alpha particle-emitting radionuclides energy profiles often mismatch with the ethically reasonable scan period for the animal. The options for the evaluation of the tissue distribution of alpha emitters currently available include specialized imaging techniques (alpha camera systems or SPECT), theranostic approaches if an equal distribution between the diagnostic and therapeutic counterpart exists, dosimetry techniques such as ex vivo tissue measurements or quantitative autoradiography. On this note, some studies deserve to be highlighted. For example, to realize imaging of actinium-225, the presence of daughter decay can be utilized to produce SPECT, by way of capturing 11% of a 218 keV emissions produced by francium-221 and the 26% 440 keV produced by bismuth-213 [101]. This method was utilized in a study using rabbits bearing VX2 hepatic tumor xenografts (Fig. 3); both the scan periods per animal and the count rates were sufficient for SPECT to display the biodistribution of an actinium-225 radiopharmaceutical [102].

**Fig. 4:**
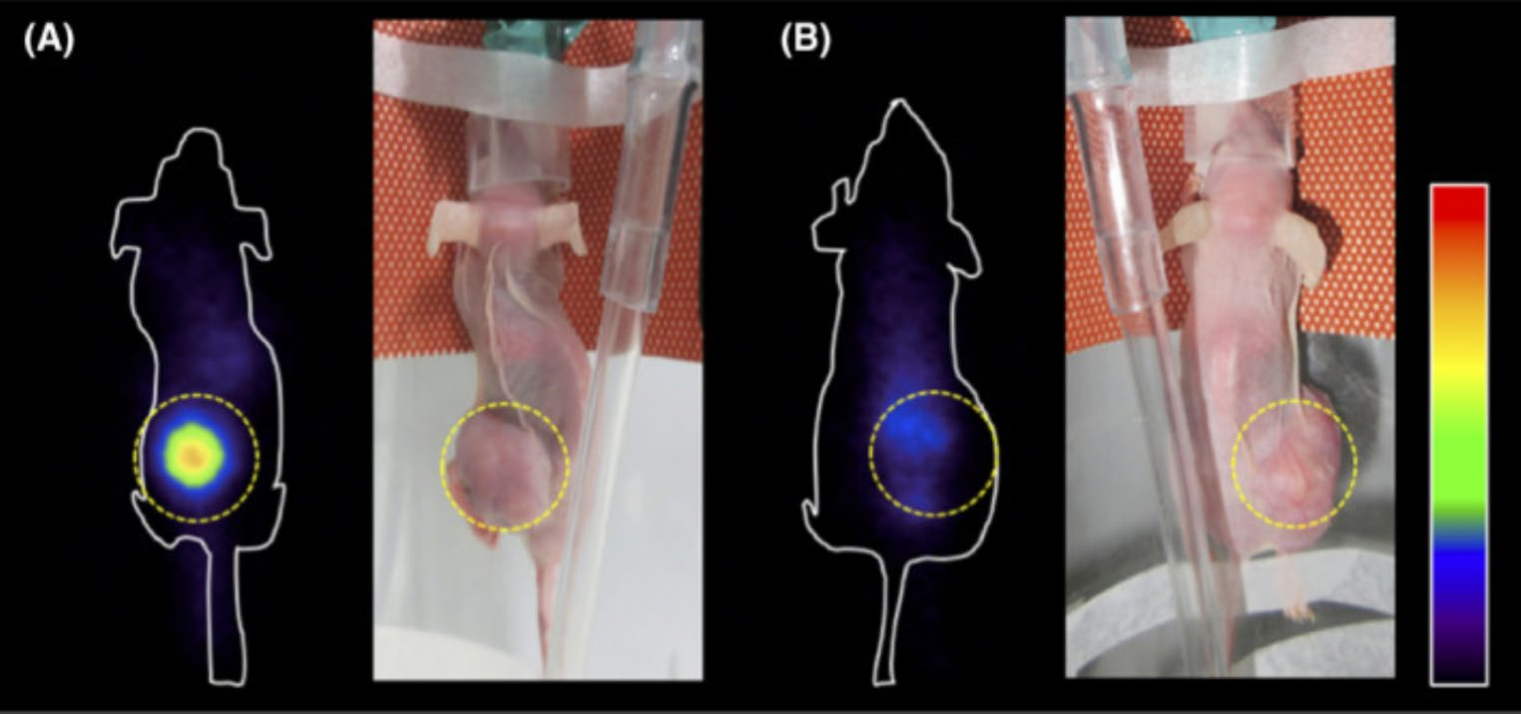
Nuclear Imaging of representative nude mice bearing PANC1 xenografts. Coronal images showing focal uptake of [^211^At]At-AAMT. Images captured at 77–92 keV energy range with a gamma camera system and low-energy all-purpose collimator. (**A**) Animal was treated with [^211^At]At-AAMT only, and (**B**) target specificity was tested for AAMT as this animal was pre-treated with a target blocking agent. (reprinted with permission from [102]

For astatine-211, the x-rays in the 77–92 keV range may be useful for SPECT imaging. An example is provided in Fig. 4. Hereby [^211^At]At-AAMT was developed for visualizing PANC1 xenografts in nude mice. Suitable images with a high local tumor-to-background ratio were captured with a gamma camera system employing the low-energy all-purpose collimator [102].

The direct quantitative imaging of ^212^Pb-Labeled radiopharmaceuticals is challenging, although a possibility with SPECT through the detection of gammas (238.6 keV) or x-rays (75–91 keV) that are emitted during the decay to bismuth-213. However, indirect imaging through the matched radioisotope pairing with lead-203 is probably the better option using SPECT imaging [103].

**Fig. 5:**
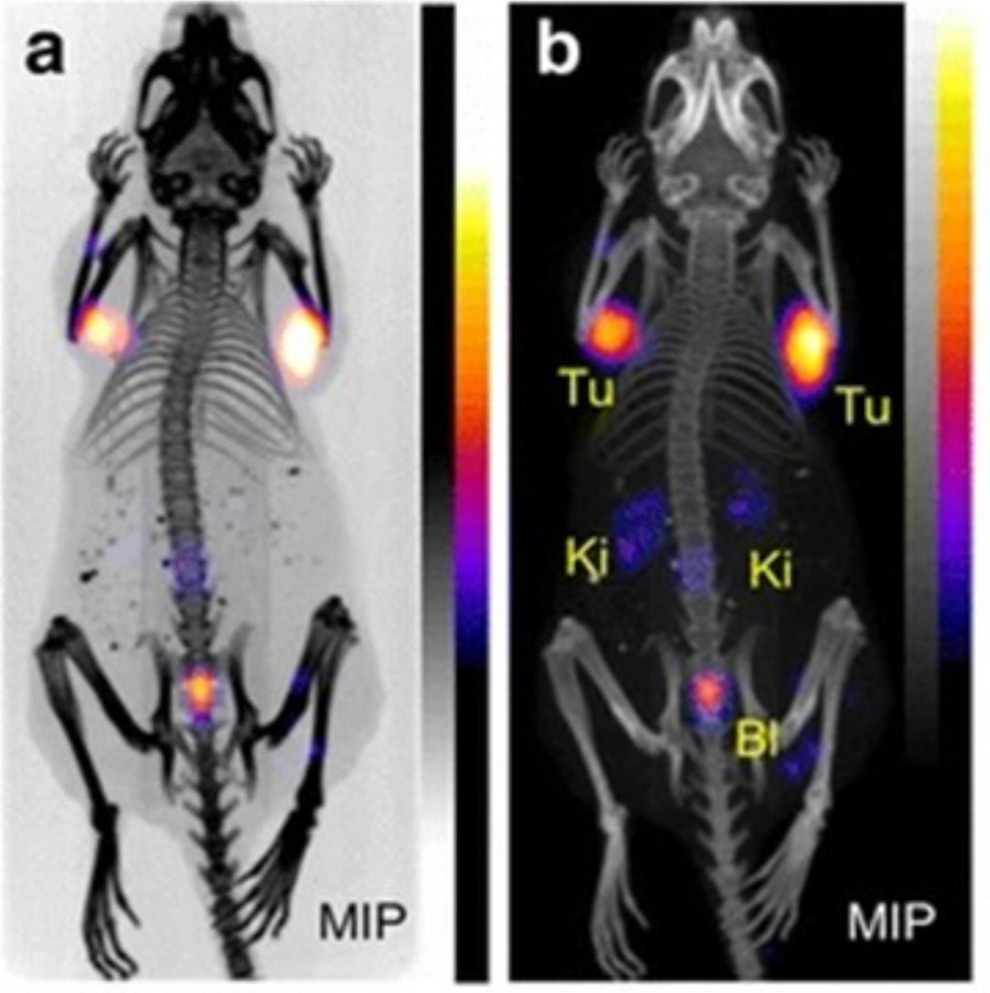
Preclinical imaging of AR42J tumor-bearing mice at 2 h following intravenous injection of [^149^Tb]Tb-DOTA-NOC (approx. 7 MBq). The microPET/CT scan shows whole body tracer distribution with distinctly high tumor (Tu) uptake; different MIPs of the same animal are displayed in **a** and **b**. Expected tracer uptake was found in kidneys (Ki) and bladder (Bl). Image reprinted with permission [76]

The terbium radioisotopes are posing a great example for elegant radiotheranostics; the combination of available Tb-radionuclides offers the most straightforward imaging possibilities. Additionally, the alpha particle-emitting radionuclide terbium-149 emits positron energy suited for PET imaging (Eβ + mean = 730 keV, β+ = 7.1%) [76] as demonstrated in Fig. 5, where the SSTR radioligand [^149^Tb]Tb-DOTA-NOC was administered intravenously which subsequently succeeded in excellent visualization of AR42J tumors in vivo using microPET/CT imaging.

The assumption that the therapeutic and diagnostic counterparts distribute equally is certainly the most convenient way to preclinically evaluate a TAT agent. Ideally, a theranostic chelator that can complex both the preferred diagnostic and therapeutic radionuclide is used; in that case only one GMP precursor needs to be developed, which has economic advantages and decreases development time. Theranostic pairs mostly used for peptide-based radiopharmaceuticals are ^68^Ga/^225^Ac-DOTA for PET/TAT applications, and for mAb constructs ^111^In/^225^Ac-DOTA for SPECT/TAT applications. However, if better PET/TAT alternatives should exist (e.g. ^89^Zr/^18^F) this should certainly be investigated.

The intricate decay properties seen for some of the TAT agents, as well as known issues with the radiochemical stability of the radiopharmaceuticals, could compromise the assumption of a similar distribution of diagnostic and therapeutic counterparts. Any mismatch in distribution can be detrimental to the therapeutic response in the patient and even lead to additional toxicity that cannot be predicted by the diagnostic counterpart. This mismatch can be investigated even further by using dual labeling studies which involves the co-injection of the diagnostic and therapeutic radiopharmaceutical together in the same animal, followed by quantification. The easiest quantification method is ex vivo biodistribution and measurement of the amount of radionuclide identified by its unique energy window.

Beyond the examples given and in alignment with the avid extension of the radiotheranostic research space, a particular focus will be expected to be on appropriate, translatable results for feasible image acquisition protocols for visualizing systemic cancer stages and monitoring therapeutic efficacy. To which extent recent innovation can meet the requirements and is capable within the preclinical setting and requires further investigations.

## Regulatory aspects

Although still non-binding recommendations, the FDA’s guidelines on the nonclinical studies and production requirements for therapeutic radiopharmaceuticals are very meaningful. These guidelines provide context on how preclinical assessment should be conducted - especially relevant for the development of new radiotheranostics. Firstly, the guidelines do consider that previous clinical experience on a ligand (e.g., the ligand was historically used for diagnostic imaging) allows for the removal of some aspects from the preclinical program [104]. In some reported instances where the evaluation of biodistribution and pharmacokinetics for a diagnostic counterpart exists, the main focus can shift to the in vivo evaluation of the therapeutic radiopharmaceutical which can focus on the tumor growth effect or animal survival rate under therapy. However, in all instances it is of crucial importance to perform a full characterization of the precursor and radiopharmaceutical with a validated quality control system. It is also very important that the stability of the therapeutic radiopharmaceutical must be fully validated. On this note, we may counter-argue that by repeating the pharmacokinetic studies with the therapeutic radiopharmaceutical may be critical as it often reveals additional pharmacological aspects that may not be under consideration during the imaging studies. This is just one of several concerns aligning with the difficulties and mismatching with the assumption of equal tissue distribution due to decay daughters and possibly lower in vivo stability of TAT agents.

The extent of daughter radionuclide(s) decays and their respective physical half-lives must also be considered as a key study design element to achieve relevant data from biodistribution experiments. Critically, the amount of radioactive but also non-radioactive materials in the dosing mixture that is evaluated in the animals should ideally mimic that of the patients [103]. It is well known that the molar activities of the diagnostic counterpart and therapeutic counterpart differ widely [105]. Furthermore, it is also recommended that during the design of a biodistribution study with a therapeutic radiopharmaceutical, all aspects of the planned clinical application should be considered. This means that any pretreatments and concomitant treatments should also be included in the study. Toxicology studies are mostly focused on radiation-induced toxicity as uniquely contributed by the ligand and radionuclide combination present in the therapeutic radiopharmaceutical [104].

When toxicology studies for radiopharmaceuticals is considered, the amount of mass for precursor and radiopharmaceutical active ingredient in the final formulation might fit the radiopharmaceutical into different categories namely < 100 µg and > 100 µg. If the compound is less than 100 µg it is proposed that the microdosing concept could be apply. In such a case it might be appropriate to do an acute toxicity study at 100 times the clinical dose (*N* = 30, 14 days) according to GLP compliant practices. As always, extensive biodistribution data should be made available from preclinical studies and dosimetry should be appropriate. Toxicity studies could then focus on risk organs and tissues identified by imaging or ex vivo biodistribution studies. If the compound is more than 100 µg the ICH guideline S9 on nonclinical evaluation for anticancer pharmaceuticals could be followed. For an interesting perspective on this matter consult Koziorowski et al. [106].

## Discussion

Inevitable access to radiotheranostics is expanding; however, pitfalls concerning alpha radionuclides accessibility and adequate usage, TAT-tailored animal models for evaluation and imaging tools, and regulatory burdens may limit the preclinical evaluation translation to new clinical procedures. Some of these aspects sparked an intense developing phase for new targeting ligands, radioisotopes, and better application methods. Basic, preclinical, and translational research may be key players for sustained progress in this field; now that the pharmaceutical industry sector and different regulatory bodies are more supportive. Therefore, this discussion will emphasize two aspects that need consideration: (1) preclinical research should be poised to give valuable recommendations on the balance between safety and efficacy and (2) it should provide information on the ideal dosing of the TAT agent.

### Balancing efficacy and safety

Balancing efficacy and toxicity are not trivial considering the impact that TAT may have on a subcellular level. **Therapeutic efficacy** requires the delivery of a dose (below the tolerable limit) of radiation to tumor cells. Elegant strategies must be used to improve efficacy as opposed to solely enhancing the amount of radiation administered and accept the trade-off to risk increases of toxicity or off-target pharmacological effects to the non-malignant tissues [1]. Drug delivery systems can be considered to shuttle radiopharmaceuticals; the role, value and limitations of available strategies were recently reviewed by the authors [107, 108].

Currently, for all preclinical development, a diagnostic partner is developed simultaneously with the α-therapeutic radiopharmaceuticals. This strategy allows for determining pharmacokinetics, biodistribution and selectivity using the diagnostic partner. The in vivo evaluation of the therapeutic partner is then employed for evaluation of toxicity, tumor growth, animal therapy tolerability and/ or monitoring (just) survival. However, we herein propose sufficient motivation for why evaluating biodistribution with a diagnostic agent alone may not fully address the clinical need, and why the investigation of biodistribution should be conducted with the therapeutic counterpart. The reported literature also shows that diagnostic radiopharmaceuticals or beta-minus-emitting radiopharmaceuticals when proven efficacious in the clinics are often “fast-tracked” for TAT therapy without any re-assuring preclinical at all. This could cause a crucial scenario as radiochemistry procedures, with the radiometal complexation, may differ significantly for alpha particle-emitting radionuclides, therefore risking and unfit use during TAT. It is also important to note that changes in affinity could result when the radiometal is exchanged. Hopefully more often, immune checkpoint inhibitors, radiosensitizers or other concomitant therapies are considered. Unfortunately, blood sampling is rarely performed to determine the stability and integrity of these therapeutic radiopharmaceuticals *in vivo.*

This emphasizes that the focus of preclinical investigations may be **more on safety concerns**. As already indicated, the in vivo stability for some of the alpha-emitters can be problematic which has been discussed, evaluated and comprehensively reviewed [15, 109]. Therefore, it is highly recommended that if any avenue of imaging exists, the TAT radiopharmaceutical should be comprehensively validated for probable concerns on, tolerability, ADME, stability or evidence for off-target distribution. As a further example, it has been demonstrated by targeting the fibroblast activation protein, the short half-life of radionuclides used for diagnostic purposes only provides a too short (and therefore unfit) window to view the complete pharmacokinetics for TAT delivery to the target [110]. However, with longer half-lives of most alpha particle emitters, the picture is much more complex and needs to consider factors such as internalization, washout and other behavior for the duration of the radionuclidic half-life.

### It is all about dose, or is it?

The efficacy and toxicity of a therapeutic radiopharmaceutical can be directly ascribed to the interplay between radiation dose delivered to a specific tissue (target or non-target) and the interplay to radiation sensitivity. The environment is further complicated by the tumor microenvironment and the interplay with the immune system. This cannot be accurately mimicked in an animal model. Small animals therefore have limited predictive value and should be viewed in perspective. Whilst studies focusing on efficacy and survival of rodents after certain treatments and combination treatments is interesting, it might not be translatable to the clinical situation.

What animal models could however contribute is to provide information on where the radiopharmaceutical accumulates and from these pharmacokinetic parameters, dosimetry can be predicted which in turn can be used to extrapolate potential efficacy and toxicity in humans. To realize these goals the focus should be on pharmacokinetic studies and preclinical toxicity studies (tailor-made for radiotherapy hence focusing on absorbed dose and expected radiation effects). Dosimetry is also extremely important. It is important to note that current translational calculations from rodent to human dosimetry is not optimal and needs further attention [111]. Alpha therapy of course has the unique constraint that preclinical imaging is not always feasible.

The presence of constraints towards imaging of the biodistribution of alpha emitters are currently solved by multiple workaround methods. Often during in vivo studies, pharmacokinetics, biodistribution and even dosimetry is extrapolated from the diagnostic partner to predict toxicity for the therapeutic partner. Rarely, biodistribution and dosimetry are done with the assistance of in vivo imaging, but a few examples has been highlighted in this review. Currently the most optimal method seems to be imaging biodistribution with a surrogate diagnostic imaging agent combined with microscale imaging (e.g., autoradiography, Cherenkov imaging or alpha-camera imaging).

Sgouros and co-workers states that combination therapy might be evaluated in preclinical models, and this is to evaluate whether the second therapeutic alters the radiosensitivity of the tumor or alters the biodistribution of the radiopharmaceutical. This is therefore not evaluating the therapeutic efficacy of a standalone radiopharmaceutical (monotherapy), but rather its behavior in the presence of another add-on therapy to highlight synergism or more importantly, the rise of unacceptable toxicity. This could also be a very useful application of preclinical studies, especially when evaluating the effect of drugs that influence the immune response of the host.

## Conclusive statements

Expanding the access to radiotheranostics in the nuclear medicine clinic face challenges such as alpha radionuclide utilization and optimal preclinical translation. The key consideration should be the optimal translation of TAT agents that have a proven balance between safety and efficacy. This is especially important because of the high cytotoxic payload of alpha-emitting radionuclides.

It is possible that the burden of proof will rely more heavily on well-designed preclinical animal studies for the future clinical translation of TAT radiopharmaceuticals. Available animals models include xenografts, allografts and PDX models and the choice of model is governed by the specific target that is investigated. Overcoming imaging constraints for alpha emitters requires innovative methods, with surrogate diagnostic agents and microscale imaging emerging as optimal. Additionally, evaluating combination therapies in preclinical models offers insights into potential synergies or toxicities, informing future clinical applications.

### Electronic supplementary material

Below is the link to the electronic supplementary material.

Supplementary Material 1

## Declarations

### Related manuscripts

The authors declare that no related manuscripts are under consideration for publication elsewhere.
