# Supplementary material for: Can current preclinical strategies for radiopharmaceutical development meet the needs of targeted alpha therapy?
Source: Eur J Nucl Med Mol Imaging. 2024 Apr 27;51(7):1965–80. doi: 10.1007/s00259-024-06719-5 (PMC11139742; doi:10.1007/s00259-024-06719-5)
Supplement: Supplementary file 1 — Supplementary Material 1 [file 259_2024_6719_MOESM1_ESM.pdf]

Supplement 1 – list of clinical trials registered on clinicltrials.gov in chronological order

Start date	Trial number	Name	Drug	Clinical trial phase
1998/02	NCT00003461	Radiolabeled Monoclonal Antibody Therapy in Treating Patients With Primary or Metastatic Brain Tumors	[^{211}At]At-Mab-81C6	Phase I/Phase 2 Completed
2000/11	NCT00014495	Chemotherapy and Monoclonal Antibody Therapy in Treating Patients With Advanced Myeloid Cancer	[^{213}Bi]Bi-labelled humanized anti-CD33 mAB HuM195	Phase I/Phase 2 Completed [32 enrolled]
2005/07	NCT00672165	Targeted Atomic Nano-Generators (Actinium-225-Labeled Humanized Anti-CD33 Monoclonal Antibody HuM195) in Patients with Advanced Myeloid Malignancies	[^{225}Ac]Ac-labelled humanized anti-CD33 mAB HuM195	Phase I – Completed [23 participants]
2012/10	NCT02575963	Lintuzumab-Ac225 in Older Acute Myeloid Leukaemia (AML) Patients	[^{225}Ac]Ac-Lintuzumab AC 225	Phase I – active, not recruiting [72 patients]
2015/11/20	NCT02581878	Safety and Tolerability of BAY1862864 Injection in Subjects With Relapsed or Refractory CD22-positive Non-Hodgkin's Lymphoma	BAY1862864	Phase 1 Completed [21 participants]
2016/01/01	NCT04833517	Prospective REgistry of Targeted RadionucLide TherapY in Patients with mCRPC (REALITY Study)	[^{225}Ac]Ac-PSMA RLT	Prospective observational [Patient registry]
2016/12	NCT02998047	A Phase I Study of Lintuzumab-Ac225 in Patients with Refractory Multiple Myeloma	[^{225}Ac]Ac-Lintuzumab AC 225	Phase I – active, not recruiting [12 patients]
2017/10/10	NCT03276572	Phase I Trial of 225Ac-J591 in Patients with mCRPC	[^{225}Ac]Ac-J591	Phase I - recruiting [31 participants]
2017/10/24	NCT03128034	211^At-BC8-B10 Before Donor Stem Cell Transplant in Treating Patients with High-Risk Acute Myeloid Leukaemia, Acute Lymphoblastic Leukaemia, Myelodysplastic Syndrome, or Mixed-Phenotype Acute Leukaemia	[^{211}At]At-BC8-B10 mAb	Suspended
2018/02/05	NCT03466216	Phase 1 Study of AlphaMedix™ in Adult Subjects With SSTR (+) NET	[^{212}Pb]Pb-DOTAM-TATE	Phase 1- recruiting [33 participants]
2018/05/22	NCT03441048	Lintuzumab-Ac225 in Combination with Cladribine + Cytarabine + Filgrastim + Mitoxantrone (CLAG-M) for Relapsed/Refractory Acute Myeloid Leukaemia	[^{225}Ac]Ac-Lintuzumab AC 225	Phase I – Recruiting [33 participants]
2018/06/13	NCT03507452	First-in-human Study of BAY2287411 Injection, a Thorium-227 Labeled Antibody-chelator Conjugate, in Patients with Tumors Known to Express Mesothelin	BAY228741	Phase 1 Completed [36 participants]
2018/12/18	NCT03724747	Study to Evaluate the Safety, Tolerability,Pharmacokinetics, and Antitumor Activity of a Thorium-227 Labeled Antibody-chelator Conjugate Alone and in Combination With	BAY 2315497	Phase 1- active, not recruiting [36 participants]

		Darolutamide, in Patients With Metastatic Castration Resistant Prostate Cancer		
2019/01/17	NCT03746431	A Phase 1/2 Study of [225Ac]-FPI-1434 Injection	$[^{225}Ac]$Ac-FPI-1434	Phase I – Recruiting [38 participants]
2020/01/01	NCT04225910	Clinical Trial of Ac225-PSMA Radioligand Therapy of Metastatic Castration-resistant Prostate Cancer	An actinium-225 containing PSMA targeting radiopharmaceutical	Early Phase I [20 participants]
2020/06/16	NCT04083183	Total Body Irradiation and Astatine-211-Labeled BC8-B10 Monoclonal Antibody for the Treatment of Non-malignant Diseases	$[^{211}At]$At-BC8-B10 mAb	Phase I/Phase 2 Recruiting [40 participants]
2020/07/02	NCT04147819	A First in Human Study of BAY2701439 to Look at Safety, How the Body Absorbs, Distributes and Excretes the Drug, and How Well the Drug Works in Participants With Advanced Cancer Expressing the HER2 Protein	BAY2701439	Phase 1 Completed [14 participants]
2020/07/08	NCT04461457	Targeted Radiation Therapy for Ovarian Cancer: Intraperitoneal Treatment With 211-astatine-MX35 F(ab')2	$[^{211}At]$At-MX35 f(ab)2	Early phase I-Completed [12 patients]
2020/08/18	NCT04506567	Fractionated and Multiple Dose 225Ac-J591 for Progressive mCRPC	$[^{225}Ac]$Ac-J591	Phase I and II [105 participants]
2020/10/29	NCT04576871	Re-treatment 225Ac-J591 for mCRPC	$[^{225}Ac]$Ac-J591	Phase I - recruiting [33 participants]
2020/11/12	NCT04644770	A Study of JNJ-69086420, an Actinium-225-Labeled Antibody Targeting Human Kallikrein-2 (hK2) for Advanced Prostate Cancer	$[^{225}Ac]$Ac-JNJ-69086420	Phase I – recruiting [50 participants]
2021/04/01	NCT04597411	Study of 225Ac-PSMA-617 in Men with PSMA-positive Prostate Cancer	$[^{225}Ac]$Ac-PSMA-617	Phase I – recruiting [30 participants]
2021/06/30	NCT04886986	225Ac-J591 Plus 177Lu-PSMA-I&T for mCRPC	$[^{225}Ac]$Ac-J591	Phase II - recruiting
2021/08/12	NCT04946370	Maximizing Responses to Anti-PD1 Immunotherapy with PSMA-targeted Alpha Therapy in mCRPC	$[^{225}Ac]$Ac-J591	Phase I/Phase II – [76 participants]
2021/12/16	NCT05219500	Targeted Alpha Therapy with 225Actinium-PSMA-I&T of Castration-resISTant Prostate Cancer	$[^{225}Ac]$Ac-PSMA I&T	Phase II – recruiting [100 participants]
2021/12/21	NCT05153772	Targeted Alpha-emitter Therapy of PRRT Naïve and Previous PRRT Neuroendocrine Tumor Patients (ALPHAMEDIX02)	$[^{212}Pb]$Pb-DOTAM-TATE	Phase 2- open label Recruitment [68 participants]
2021/12/22	NCT05283330	Safety and Tolerability of ^{212}Pb-DOTAM-GRPR1 in Adult Subjects With Recurrent or Metastatic GRPR-expressing Tumors	$[^{212}Pb]$Pb-DOTAM-GRPR1	Phase 1- recruiting [30 participants]
2022/03/24	NCT05477576	Study of RYZ101 Compared with SOC in Pts w Inoperable SSTR+ Well-differentiated GEP-NET that has Progressed following 177Lu-SSA Therapy (ACTION-1)	$[^{225}Ac]$Ac-DOTA-TATE	Phase III – recruiting [288 participants]
2022/04/20	NCT05363605	A Study of $[^{225}Ac]$-FPI-1966 in Participants with Advanced Solid Tumours	$[^{225}Ac]$Ac-FPI-1966	Phase I and Phase II Completed [6 participants]

2022/06/02	NCT05204147	A Phase I Study of Actinium-225 Labelled Humanized Anti-CEA M5A Antibody in Patients with CEA Producing Advanced Colorectal Cancer	[^{225}Ac]Ac-DOTA-anti-CEA monoclonal Antibody M5A	Early Phase I – [18 participants]
2022/07/21	NCT05496686	Targeted Alpha Particle Radiotherapy for Metastatic Uveal Melanoma	[^{225}Ac]Ac-MTI-201	Phase I - recruiting [16 participants]
2022/07/21	NCT05496686	Targeted Alpha Particle Radiotherapy for Metastatic Uveal Melanoma	[^{225}Ac]Ac-MTI-201	Phase I -recruiting [16 participants]
2022/10/10	NCT05595460	Study of RYZ101 in Combination with SoC in Subjects With SSTR+ ES-SCLC	[^{225}Ac]Ac-DOTA-TATE	Phase I – recruiting [31 participants]
2022/11/22	NCT05363111	Phase 1 Trial of 111Indium/225Actinium-DOTA-Daratumumab in Patients With Relapsed/Refractory Multiple Myeloma	[^{225}Ac]Ac-DOTA-Daratumumab	Phase I – Not yet recruiting [15 participants]
2023/02/07	NCT05605522	A Study of [^{225}Ac]-FPI-2059 in Adult Participants with Solid Tumours	[^{225}Ac]Ac-FPI-2059	Phase I-recruiting [42 participants]
2023/03/15	NCT05720130	Dose Escalation and Efficacy Study of 212Pb-ADVC001 in Patients With Metastatic Castration Resistant Prostate Cancer.	[^{212}Pb]Pb-ADVC001	Phase I and Phase II Recruiting [6 participants]
2023/09/20	NCT06052306	A Study to Learn How Safe the Study Treatment Actinium-225-macropa-pelgifatamab (BAY3546828) is, How it Affects the Body, How it Moves Into, Through and Out of the Body, and About Its Anticancer Activity in Men With Advanced Metastatic Castration-resistant Prostate Cancer (mCRPC)	[^{225}Ac]Ac-Macropa-pelgifatamab	Phase I- recruiting [140 participants]
2024/01/27	NCT04466475	Radioimmunotherapy (211At-OKT10-B10) and Chemotherapy (Melphalan) Before Stem Cell Transplantation for the Treatment of Multiple Myeloma	[^{211}At]At-OKT10-B10	Phase I Recruiting [30 patients]
2024/02/01	NCT04579523	^{211}At-OKT10-B10 and Fludarabine Alone or in Combination With Cyclophosphamide and Low-Dose TBI Before Donor Stem Cell Transplant for the Treatment of Newly Diagnosed, Recurrent, or Refractory High-Risk Multiple Myeloma	[^{211}At]At-OKT10-B10	Phase I Recruiting [30 patients]
